# Comparison of Ecological Micro-Expression Recognition in Patients with Depression and Healthy Individuals

**DOI:** 10.3389/fnbeh.2017.00199

**Published:** 2017-10-17

**Authors:** Chuanlin Zhu, Xinyun Chen, Jianxin Zhang, Zhiying Liu, Zhen Tang, Yuting Xu, Didi Zhang, Dianzhi Liu

**Affiliations:** ^1^Department of Psychology, School of Education, Soochow University, Suzhou, China; ^2^Suzhou Psychiatric Hospital, Suzhou, China

**Keywords:** micro-expression recognition, ecological, depression, negative bias, context

## Abstract

Previous studies have focused on the characteristics of ordinary facial expressions in patients with depression, and have not investigated the processing characteristics of ecological micro-expressions (MEs, i.e., MEs that presented in different background expressions) in these patients. Based on this, adopting the ecological MEs recognition paradigm, this study aimed to comparatively evaluate facial ME recognition in depressed and healthy individuals. The findings of the study are as follows: (1) background expression: the accuracy (ACC) in the neutral background condition tended to be higher than that in the fear background condition, and the reaction time (RT) in the neutral background condition was significantly longer than that in other backgrounds. The type of ME and its interaction with the type of background expression could affect participants’ ecological MEs recognition ACC and speed. Depression type: there was no significant difference between the ecological MEs recognition ACC of patients with depression and healthy individuals, but the patients’ RT was significantly longer than that of healthy individuals; and (2) patients with depression judged happy MEs that were presented against different backgrounds as neutral and judged neutral MEs that were presented against sad backgrounds as sad. The present study suggested the following: (1) ecological MEs recognition was influenced by background expressions. The ACC of happy MEs was the highest, of neutral ME moderate and of sadness and fear the lowest. The response to the happy MEs was significantly shorter than that of identifying other MEs. It is necessary to conduct research on ecological MEs recognition; (2) the speed of patients with depression in identifying ecological MEs was slower than of healthy individuals; indicating that the patients’ cognitive function was impaired; and (3) the patients with depression showed negative bias in the ecological MEs recognition task, reflecting the lack of happy ME recognition ability and the generalized identification of sad MEs in those patients.

## Introduction

Micro-expressions (MEs) are very fast (1/25–1/2 s) facial expressions, MEs contribute to revealing individuals’ emotions that they attempt to conceal (Ekman, 2003; Matsumoto and Hwang, 2011; Yan et al., 2013). Ekman and Friesen (1974) developed the first ME recognition test, the Brief Affect Recognition Test (BART). In this test, various ME images (happiness, sadness, fear, anger, disgust and surprise) are presented (1/100–1/25 s), participants are asked to complete an emotional classification task, and then the corresponding accuracy (ACC) is analyzed. Although the BART laid the foundations for further research, it also has some shortcomings. First, it is difficult to measure real ME recognition with the BART, due to the lack of masking after the target stimulus, which may extend the time of processing the target stimulus, resulting in the processing being influenced by the visual aftereffects. Second, in the BART, each ME is presented independently, while no forward and backward expressions are presented; hence, participants cannot know the background information associated with the ME, which has led to questioning the ecological validity of the test.

In order to overcome these shortcomings, Matsumoto et al. (2000) developed an improved test, the Japanese and Caucasian BART (JACBART), based on the BART. In the JACBART, a non-neutral face expression image (target stimulus) is embedded in a neutral face expression video (mask stimuli), with a time of 1 s. The identity of the people in the target and mask stimuli are controlled. The participants’ task is to identify the emotion conveyed by the target stimuli. Numerous studies (Hall and Matsumoto, 2004; Russell et al., 2006; Matsumoto and Hwang, 2011) have reported that the JACBART has good reliability and validity; therefore, the test is being widely used. Though the JACBART succeeded in eliminating the influence of visual aftereffects, it only examined ME processing in the neutral (non-emotional) context, and did not examine it in the emotional context, such as pleasure, sadness and fear. However, in real life, MEs are present in emotional expressions as well. Does ME recognition differ in emotional and non-emotional contexts? That is to say, is ME recognition influenced by the types of context?

Aiming to solve this problem, based on the JACBART, Zhang et al. (2014) were the first to explore the role of neutral, sad and happy contexts in the ME recognition task. The results showed that the ACC of recognizing all MEs was decreased in the sad context compared to the neutral and happy contexts, which suggests that participants’ performance in the ME recognition task is influenced by the context. This study was a further refinement of the JACBART. However, the types of context that were adopted in Zhang et al.’s (2014) study were still limited. Soon after that, Zhang et al. (2017) examined the ME recognition characteristics of college students in fearful, sad, disgusting, angry, surprised and happy contexts. The results showed that the main effects of the fearful, sad, disgusting and angry context were significant, while those of surprise and happiness were not significant; on the basis of these results, Zhang et al. (2017) established an ecologically valid ME recognition test. Ecological MEs are MEs that occur in real life, rather than MEs only accompanying neutral expressions. The MEs in Zhang et al.’s (2017) study were ecologically valid; therefore, they are considered to be ecological MEs.

Previous studies have shown that, in addition to sex (Hall and Matsumoto, 2004), age (Mill et al., 2009; Hurley et al., 2014) and personality (Hurley et al., 2014), depression (Liu et al., 2012; Gollan et al., 2015b; Kerestes et al., 2016; Milders et al., 2016) can affect one’s performance when recognizing ordinary facial expressions. Depression is one of the most common mental illness (Bocharov et al., 2017); the official website of WHO reports that the global prevalence of patients with depression exceeds 300 million. Unlike the usual mood swings or emotional responses to daily challenges, long-term moderate or severe depression can lead to serious health issues, and in the most severe cases, depression can lead to suicide. Every year, about 800,000 people commit suicide due to depression, and suicide is the second leading cause of the death in people aged 15–29 years. Many researchers have studied the recognition characters of ordinary facial expressions in patients with depression; the results showed that the patients showed obvious negative bias when processing ordinary facial expressions (Dai and Feng, 2012; Gollan et al., 2015a; Jaworska et al., 2015; Fonseka et al., 2016). Compared with happy and neutral expressions, the patients were more sensitive to sad expressions (Maniglio et al., 2014; Zhang et al., 2016), and they tended to judge happy expressions as neutral (Bocharov et al., 2017), while judging neutral expressions as sad (Maniglio et al., 2014; Fonseka et al., 2016). In addition, researchers found that patients with depression can accurately identify ordinary facial expressions (Gollan et al., 2008; Robinson et al., 2015), but their reaction time (RT) were longer than that of healthy individuals (Başgöze et al., 2015). However, in addition to ordinary facial expressions, there are many MEs in our daily life.

Compared with ordinary expressions, ME recognition has its particularities. First, recognizing ME requires higher sensitivity and recognition ability (Matsumoto and Hwang, 2011). Therefore, there may be differences in the characteristics of ordinary expressions and MEs in patients with depression. Second, ME recognition ability is associated with discerning ability (Hurley et al., 2014; Yin et al., 2016) and social ability (Matsumoto and Hwang, 2011), so the defects in recognizing MEs could reflect defects in discerning and social skills, to some degree. However, the negative bias showed in previous studies was based on adopting ordinary expressions as stimuli. To our knowledge, there has been no study examining the ME recognition characteristics of patients with depression. Based on this discussion, employing the ecological MEs recognition test established by Zhang et al. (2017), this four (contexts: happy, neutral, sad and fearful) × 4 (ME: happy, neutral, sad and fearful) × 2 (group: depression and control group) study aimed to explore the ecological MEs recognition characteristics of patients with depression. Based on previous studies and the characteristics of MEs, we hypothesized that: (1) background expressions affect participants’ recognition of MEs; (2) compared with healthy individuals, the patients with depression would show lower ACC and longer RT while completing the ecological MEs recognition task; and (3) patients with depression would also show a negative bias in the ecological MEs task.

## Materials and Methods

### Subjects

Thirty unmedicated patients (21 females) with a first episode of depression, diagnosed with a current depression according to the Diagnostic and Statistical Manual (DSM-IV), were selected from Suzhou Guangji Hospital. Inclusion criteria are: (1) no head trauma experience; (2) no drug and other material abuse experience; (3) female subjects are not breast-feeding or pregnancy; (4) no anxiety disorder, bipolar disorder and other mental illness; (5) between 20–60 years old; and (6) the degree of education is junior high school and above. Thirty healthy individuals (control group, 21 females) were enrolled. The two groups were matched in age, education and handedness. All subjects were right hand, normal or corrected to normal eyesight visual acuity. All subjects provided written informed consent, which was in accordance with the Declaration of Helsinki (1991) before the experiment, which was approved by the Suzhou Psychiatric Hospital Ethics Committee. Participants received 50 RMB for participation.

The depression level of all subjects was measured using the Chinese version of the Beck Depression Inventory II (BDI-II; Beck et al., 1997), revised by Wang et al. (2011). The revised version’s internal consistency coefficient Cronbach α is 0.94. The score of <14 points means no depression, ≥14 points means depression, the scores of all control group members’ were below 14, while all patients scores were higher than 14. The two groups were matched in age, education and handless. The basic information of all subjects is shown in Table 1.

**Table 1:** Characteristics of the patient and control groups (*N* = 30).

	Patient (M ± SD)	Control (M ± SD)	*t*
Mean age	36.93 ± 11.86	37.60 ± 12.06	1.29
Education time	12.96 ± 3.09	13.33 ± 3.08	0.78
BDI	24.83 ± 6.88	7.63 ± 3.63	14.56***

Note: “***” stands for “*p* < 0.001”. BDI, Beck Depression Inventory; M, mean; SD, standard deviation.

### Stimuli and Procedure

**Figure 1:**
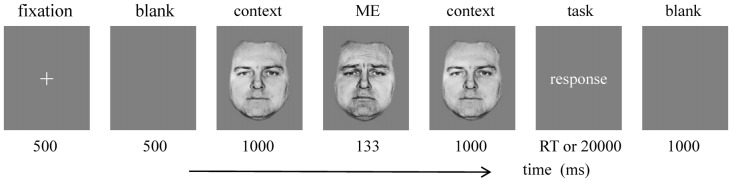
Illustration of one experimental trial.

Forty grayscale images (338 × 434 pixels) of 10 models (five females) with facial expressions of happiness, neutral, sadness and fear were selected from Ekman’s Pictures of Facial Affect (POFA; Ekman and Friesen, 1976). The experimental program was programmed with E-prime 2.0. The procedure was consisted of four blocks, while each block comprised of 40 trials, a total of 160 trials. As shown in Figure 1, each trial started with a 500 ms white fixation cross, followed by a blank (500 ms), the expression context (1000 ms), the target expression (133 ms), next, the same context was presented (1000 ms). After that, the labels of the four target expressions (happiness, neutral, fear and sadness) were presented, subjects were required to discriminate the target expression. They were instructed to press the “D” key with their left middle finger, if the target expression was happiness, key “F” with their left index finger when neutral, key “J” with their right index finger when sadness, key “K” with their right index finger when fear. The images used in each trial comes from the same model. Participants were told to complete the task as accurately as possible (up to 20,000 ms). Finally, a blank (1000 ms). The block design has been adopted in this study, only one type of contexts (neutral, sad, happy and fear) was adopted in each block. All stimuli were presented in the center of the screen.

This study was conducted in a sound attenuated room, subjects sat in front of a 17-inch CRT monitor, with a resolution of 1280 × 1024 pixels and a refresh rate of 75 Hz, at a distance of 70 cm. In order to ensure subjects fully understood the flowchart, 16 practice trials were provided before the formal test. Feedback was provided for each trial in the practice phase, while no feedback was provided in the formal test. The flowchart of the practice trials were the same as the form test. In order to minimize fatigue effects, all subjects were asked to rest 2 min after each block.

### Data Recording and Analysis

The data in this study was collected by E-prime 2.0. All statistical analyses were performed using SPSS 16.0, *post hoc* testing was conducted using the Bonferroni correction, while *p* values were corrected by Greenhouse-Geisser method.

## Results

### Indicator 1: The ACC of Recognizing MEs

The results of the one-sample *t*-test showed that the ACC of recognizing all MEs was significantly (*p*s < 0.01) higher than chance (0.25), indicating that the ACC was not the result of random guessing. For the measures of ACC in recognizing MEs, three-way repeated-measures ANOVA was performed, with the type of context and ME as the within-subject factors and group (patients vs. controls) as the between-subjects factor. The results showed that the main effect of context was significant (*F*_(3,174)_ = 2.953, *p* = 0.034, η^2^ = 0.048), the ACC under the neutral background expression condition tended to be higher than that under the fear background expression condition (*p* = 0.068), and the ACC under any other two background expression conditions showed no significant difference (*p*s > 0.327), indicating that the individuals’ ecological MEs recognition ACC was affected by the type of background expression. The main effect of ME was significant (*F*_(3,174)_ = 52.795, *p* < 0.001, η^2^ = 0.477); the *post hoc* analysis revealed that the ACC under the happy ME condition was higher than that under the neutral, sad and fear ME conditions (*p* < 0.001), while the ACC under the neutral ME condition was higher than that under the sad (*p* = 0.001) and fear (*p* < 0.001) ME conditions. Indicating participants’ ecological MEs recognition ACC was affected by the type of ME. The main effect of group was not significant (*F*_(1,58)_ = 0.121, *p* = 0.729, η^2^ = 0.002). The interaction of context with ME was significant (*F*_(9,522)_ = 24.062, *p* < 0.001, η^2^ = 0.293), the interaction of context with group (*F*_(3,174)_ = 0.727, *p* = 0.537, η^2^ = 0.012), ME with group (*F*_(3,174)_ = 1.340, *p* = 0.263, η^2^ = 0.023), and context with ME with group (*F*_(9,522)_ = 1.318, *p* = 0.224, η^2^ = 0.022) were not significant.

As the interaction effect of context with ME was significant, a simple effect analysis was conducted and the results were as follows. In the neutral background expression condition, the ACC of happy and neutral MEs was higher (*p*s < 0.001) than that of sad and fear MEs. In the happy background expression condition, the ACC of happy MEs was higher (*p*s < 0.001) than that of neutral, sad and fear MEs, while the ACC of fear MEs was higher (*p* = 0.037) than that of sad MEs. In the sad background expression condition, the ACC of happy MEs was higher than that of neutral (*p* < 0.001), sad (*p* = 0.015) and fear (*p* < 0.001) MEs, while the ACC of sad MEs was higher (*p*s < 0.001) than that of neutral and fear MEs. In the fear background expression condition, the ACC of happy MEs was higher than that of neutral (*p* = 0.016), sad (*p* < 0.001) and fear (*p* = 0.001) MEs, while the ACCs of neutral and fear MEs were higher (*p*s < 0.001) than that of sad MEs.

In conclusion, the type of context tended to influence individuals’ ACC of recognizing MEs, the type of ME significantly influenced the individuals’ ACC, depression had no significant influence on the individuals’ ACC of recognizing MEs. Additionally, the individuals’ ACC was significantly influenced by the interaction effect of context with ME.

### Indicator 2: The RT of Recognizing MEs

For the measures of RT of recognizing MEs, three-way repeated-measures ANOVA was performed, with the type of context and ME as the within-subject factors and group (patients vs. controls) as the between-subjects factor. The results showed that the main effect of context was significant (*F*_(3,174)_ = 11.241, *p* < 0.001, η^2^ = 0.162), the *post hoc* analysis showed that the RT under the neutral background expression condition was longer than those under the happy (*p* < 0.001), sad (*p* = 0.026), and fear (*p* < 0.001) background expression conditions; the RTs under any other two background expression conditions showed no significant difference (*p*s > 0.379). This indicated participants’ ecological MEs recognition RT was affected by the type of background expression condition. The main effect of ME was significant (*F*_(3,174)_ = 5.753, *p* = 0.002, η^2^ = 0.090), the *post hoc* analysis revealed that the RT under the happy ME condition was shorter than those under the neutral (*p* = 0.010), sad (*p* < 0.001), and fear (*p* = 0.002) ME conditions; the RT under any other two ME conditions showed no significant difference (*p*s > 0.05). This indicated participants’ ecological MEs recognition RT was affected by the type of ME. The main effect of group was significant (*F*_(1,58)_ = 9.498, *p* = 0.003, η^2^ = 0.141), the patients responded slower than did healthy individuals, suggesting that there were defects in the recognition speed of MEs in patients. The interaction of context with ME was significant (*F*_(9,522)_ = 5.345, *p* < 0.001, η^2^ = 0.084). The interaction of context with group (*F*_(3,174)_ = 1.185, *p* = 0.317, η^2^ = 0.020), ME with group (*F*_(3,174)_ = 0.428, *p* = 0.733, η^2^ = 0.007), and context with ME with group (*F*_(9,522)_ = 1.036, *p* = 0.402, η^2^ = 0.018) were not significant.

As the interaction effect of context with ME was significant, a simple effect analysis was conducted and the results are as follows: (1) happy ME: the patients’ RT under different background expression conditions showed significant difference (*F*_(3,174)_ = 5.41, *p* = 0.001); a *post hoc* analysis revealed that the RT under any two background expression conditions showed no significant difference (*p*s > 0.05). The healthy individuals’ RT under different background expression conditions showed no significant difference (*F*_(3,174)_ = 1.04, *p* = 0.375); (2) neutral ME: the patients’ RT under different background expression conditions showed significant difference (*F*_(3,174)_ = 4.31, *p* = 0.006), the RT under the happy background expression condition was longer (*p* = 0.017) than that under the fear background expression condition, suggesting it was difficult for patients to recognize MEs under the happy background expression condition. The healthy individuals’ RT under different background expression conditions showed significant difference (*F*_(3,174)_ = 3.57, *p* = 0.015), the RT under the happy background expression condition was longer (*p* = 0.002) than that under the fear background expression condition, suggesting that the happy background expressions decreased the speed of the healthy individuals in recognizing MEs compared to fear background expressions; and (3) sad ME: the patients’ RT under different background expression conditions showed no significant difference (*F*_(3,174)_ = 1.42, *p* = 0.238). The healthy individuals’ RT under different background expression conditions showed significant difference (*F*_(3,174)_ = 4.54, *p* = 0.004); the RT under the sad background expression condition was longer than those under the neutral (*p* = 0.002) and fear (*p* = 0.013) background expression conditions. (4) Fear ME: the patients’ RT under different background expressions showed significant difference (*F*_(3,174)_ = 8.44, *p* < 0.001); the RT under the happy background expression condition was longer than those under the neutral (*p* = 0.015) and fear (*p* = 0.048) background expression conditions, suggesting that the happy background expressions decreased the speed of the patients in recognizing fear MEs. The healthy individuals’ RT under different background expressions showed no significant difference (*F*_(3,174)_ = 1.94, *p* = 0.124). See Table 2 for details.

**Table 2:** The simple effect analysis results of the reaction time (RT) of patients with depression and healthy individuals under different conditions.

ME	Group	RT under different context
Neutral	Patients with depression	Happy > fear
	Healthy individuals	Happy > fear
Sad	Healthy individuals	Sad > neutral, fear
Fear	Patients with depression	Happy > neutral, sad

In conclusion, the influence of different background expressions on RT of individuals recognizing MEs showed significant difference, the type of ME significantly influenced individuals’ RT, and the patients’ RT was longer than that of healthy individuals. Additionally, individuals’ RT was significantly influenced by the interaction effect of context with ME.

### Indicator 3: Negative Bias

When complete the ecological MEs recognition task, there were four options (happy, neutral, sad and fear) for participants to choose; the probability of any one of the options to be selected was 0.25. Misjudgment refers to identifying one ME as another. One-sample *t*-test (test value = 0.25) was conducted, with the misjudgment mode that judge happy MEs as neutral, and judge neutral MEs as sad under different background expression conditions as the dependent variable, and the results are shown in Table 3.

**Table 3:** The patients’ misjudgment of the happy and neutral micro-expressions (*df* = 29).

Misjudgment mode	*M*	*SD*	*t*	*p*
Neutral-happy-neutral	0.080	0.132	−7.035	0.000
Sad-happy-neutral	0.097	0.161	−5.224	0.000
Fear-happy-neutral	0.130	0.137	−4.803	0.000
Sad-neutral-sad	0.157	0.230	2.298	0.029
Happy-neutral-sad	0.073	0.166	−0.180	0.858
Fear-neutral-sad	0.240	0.222	−0.246	0.807

*Note: “sad-happy-neutral” signifies that participants judged the happy micro-expression (ME) under the sad expression condition as neutral. The same applies for the other conditions. M, mean; SD, standard deviation*.

## Discussion

Previous studies have explored the characteristics of facial expression processing of patients with depression using ordinary facial expressions as stimuli. No study has yet reported on those patients’ ecological MEs processing characteristics, although ecological MEs (vs. ordinary facial expressions) are more consistent with real-life expressions and could better elucidate the facial expression processing characteristics in patients with depression. By adopting an ecological MEs recognition paradigm (Zhang et al., 2017), the present study explored the ecological MEs recognition characteristics of patients with depression for the first time.

Individuals’ performance in ecological MEs recognition was affected by background expressions. First, in terms of ACC, the results of this study showed that the ACC under the neutral background expression condition tended to be higher than that under the fear background expression condition, indicating that ignoring the influence of background expressions (Ekman and Friesen, 1974) or taking into consideration only the influence of neutral background expressions (Matsumoto et al., 2000) is inappropriate. When studying individuals’ ME recognition characteristics, the role of different background expressions should be fully considered. Second, in terms of RT, the results showed that individuals’ RT was longer when recognizing MEs under the neutral background expression condition than under the happy, sad and fear background expression conditions. This may be because neutral background expressions conveyed less emotional information and could not effectively promote individuals’ processing of MEs compared with the other three background expression conditions, resulting in a decrease in individuals’ processing speed while recognizing MEs under this condition. In short, both the ACC and RT of individuals’ recognition of MEs were influenced by background expressions, which confirmed our hypothesis. Therefore, the role of different background expressions should be fully considered when investigating individuals’ performance in recognizing ecological MEs.

Individuals’ performance in ecological MEs recognition was affected by the type of ME. In the present study, happy, neutral, sad, and fear MEs were studied and the results showed that, in terms of ACC, happy ME had the highest, neutral MEs had moderate and sad and fear MEs had the lowest ACC, and the ACC of recognizing sad and fear MEs showed no significant difference. This indicated that happy MEs are easier to recognize than the other studied MEs, which is consistent with previous study findings (Schaefer et al., 2010; Kujawa et al., 2014; Kluczniok et al., 2016). In terms of RT, the RT of recognizing the happy ME were significantly shorter than the RTs of recognizing neutral, sad and fear MEs, while the RT of recognizing the latter three MEs showed no significant difference, suggesting individuals were more sensitive to happy ME than to the others.

Individuals’ ACC and RT were affected by both the type of background expression and by ME alone and by the interaction effect of those two factors. For example, compared with sad and fear MEs, individuals’ ACC of recognizing the happy ME under the neutral background expression condition was higher. When recognizing the neutral ME, the recognition speed under the fear background expression condition was quicker than that under the happy background expression condition. It is suggested that the influence of background expression and ME should not be considered in isolation when exploring individuals’ performance in recognizing ecological MEs. In addition, it should be noted that when the type of background expressions and ME are congruent, the expression recognition task may be considered to be an ordinary, as opposed to ecological, expression recognition task and these two tasks should be distinguished. For example, individuals’ ACC of recognizing neutral expression was higher than the ACCs of recognizing sad and fear expressions, under the neutral background expression condition; however, under this condition, the neutral expression is an ordinary expression, while the sad and fear expressions are considered to be ecological MEs. The difference between the ACC of recognizing neutral and sad/fear expressions was not necessarily caused by the interaction between the type of background expression and ME, but it was likely caused by the difference between the ordinary expression and the ME.

Individuals’ performance in ecological MEs recognition was affected by the presence of depression. In terms of ACC, there was no significant difference between the patients and healthy individuals, which was consistent with the findings of patients processing ordinary facial expressions (Leppänen et al., 2004; Meyers et al., 2015; Robinson et al., 2015), which indicated that individuals’ ACC of recognizing ecological MEs was not affected by the presence of depression. In addition, a review (Bourke et al., 2010) revealed that compared with healthy individuals, patients with depression allocate more (less) attention resources to sad (pleasant) facial expressions, while processing ordinary facial expressions, but numerous studies have shown that the ACC of recognizing ordinary facial expressions showed no significant difference between these two groups, which supports the results of the present study to some extent. In terms of RT, the patients’ RT was longer than that of healthy individuals, which was consistent with the results of patients processing ordinary facial expressions (Wu et al., 2016; Zhang et al., 2016). However, Dai et al. (2016) found that the RT of patients with depression and healthy individuals processing neutral expression showed no significant difference, while the patients’ RT of processing sad expression was shorter than that of healthy individuals. The difference between Dai et al. (2016) and the present study may have been caused by the different experimental task. The task in the Dai et al. (2016) study was an emotional valence evaluation task, and had no time limitations, while the participants in this study were asked to complete an emotional labeling task within a limited time. Researchers could replace the experimental task in this study with an emotional valence evaluation task in the future, so as to further compare the characteristics of processing speed of patients with depression completing different types of facial expression processing tasks. The results of this study show that RT could reflect the difference between patients with depression and healthy individuals, when comparing their ecological MEs recognition characteristics, indicating that RT is an indicator more sensitive than ACC. Our second hypothesis was partially confirmed. In addition, the present study further confirmed that the presence of clinical depression affects the RT of the patients’ while performing cognitive task.

A large number of previous studies (Dai and Feng, 2012; Li et al., 2015; Fonseka et al., 2016; Zhang et al., 2016) showed that when processing ordinary expressions, patients with depression showed an obvious negative bias; they tended to judge happy MEs as neutral (Bourke et al., 2010; Bocharov et al., 2017). The present study showed that under three different background expression conditions (neutral, sad and fear), patients with depression tended to judge happy MEs as neutral, which was consistent with the findings of these previous studies. These results also show that the patients judging happy MEs as neutral is a stable phenomenon, which does not depend on the background expression. Meanwhile, previous studies (Gollan et al., 2008; Fonseka et al., 2016) have shown that patients with depression tend to judge neutral expressions as sad. The present study showed that patients tended to misjudge neutral MEs under the sad background expression condition as sad, and the misjudge probability reached a statistically significant level. Therefore, our third hypothesis was confirmed. Based on the results of this study, we could draw the conclusion that there are two types of negative bias when patients with depression recognize ecological MEs: first, the ME recognition of patients with depression depends on negative background expressions. Judging neutral MEs under sad background expression conditions as sad suggests that the patients have lower emotional self-control ability, which is affected by the negative background (or environment). Second, the patients judging happy MEs as neutral under any background expression condition reflects their lack of pleasant experience (Yang and Jones, 2008; Stuhrmann et al., 2013). These two types of negative bias may be markers of depression. Our findings showed the unique characteristics of ME recognition in patients with depression and demonstrated the value of studying ecological MEs.

Based on previous studies on processing of ordinary facial expressions in patients with depression, by adopting the ecological MEs recognition paradigm, the present study explored the ME recognition characteristics in patients with depression for the first time. This study differed from ordinary facial expression tasks (previous studies) as it extended the task to ecological MEs, which may contribute to furthering the understanding of the processing characteristics of facial expressions in patients with depression. Compared with the classical ME recognition paradigm (only considering the role of neutral context in expression recognition), the ecological MEs recognition paradigm has been adopted in this study, which could simultaneously compare the influence of happy, sad and fearful contexts in ME recognition. Our paradigm is closer to MEs as they appear in daily life; thus, it has superior ecological validity. It both contributes to attaining an in-depth understanding of the role of context in ecological MEs recognition and may be used as an adjunct diagnostic indicator for depression. Though the diagnosis of depression is eminently clinical, having solid psychiatric, biochemical, and neurofunctional underpinnings, if the ecological MEs recognition characteristics of patients, as revealed in this study, could be combined with existing psychiatric and other indicators, the objectivity and comprehensiveness of depression diagnosis could be further enhanced.

Previous studies indicated that patients with schizophrenia showed abnormal emotional experience and facial expression recognition (Sanchez et al., 2014; Zhu et al., 2016); they experienced more negative and less positive emotion than did healthy individuals. Meanwhile, the ability of recognizing positive expressions was weaker in male patients with schizophrenia than in healthy individuals, while the ability of recognizing both positive and negative emotions was similar in female patients with schizophrenia and in healthy individuals. Based on what was mentioned above, the recognition characteristics of ecological MEs in patients with schizophrenia could be explored and, then, compared with their processing characteristics of ordinary facial expressions; thus, this line of research could contribute to further the understanding of processing characteristics of facial expressions in patients with schizophrenia. Additionally, the ecological MEs recognition characteristics of patients with depression and patients with schizophrenia could be compared.

## Conclusion

Adopting the ecological MEs recognition paradigm, the present study revealed the ecological MEs recognition characteristics of patients with depression for the first time and extended the scope of facial expression processing in patients with depression. The results showed that: (1) the ecological MEs recognition of patients with depression could be influenced by background expressions and it is necessary to conduct research on ecological MEs recognition; (2) patients with depression showed defects in cognitive function when processing ecological MEs, manifested as a slower RT compared to the RT of healthy individuals; and (3) patients with depression had a negative bias when performing the ecological MEs recognition task; they tended to judge happy MEs under different background expression conditions, and judge the neutral ME under the sad background expression condition as sad, which may reflect their deficit in recognizing positive emotions and their tendency to generalize sad emotions.

## Author Contributions

CZ, XC, JZ, ZL, ZT, YX, DZ and DL conceived the study and coordinated the experiments. XC, ZL, YX, DZ and ZT performed the experiment. CZ and XC analyzed the data. CZ wrote the manuscript. CZ, JZ and DL revised the manuscript, all authors read and approved the final manuscript.

## Conflict of Interest Statement

The authors declare that the research was conducted in the absence of any commercial or financial relationships that could be construed as a potential conflict of interest.

## Abbreviations

ACC: accuracy
ME: micro-expression
RT: reaction time.
